# Inter-individual variability in structural brain development from late childhood to young adulthood

**DOI:** 10.1016/j.neuroimage.2021.118450

**Published:** 2021-08-03

**Authors:** Kathryn L. Mills, Kimberly D. Siegmund, Christian K. Tamnes, Lia Ferschmann, Lara M. Wierenga, Marieke G.N. Bos, Beatriz Luna, Chun Li, Megan M. Herting

**Affiliations:** a Department of Psychology, University of Oregon, USA; b PROMENTA Research Center, Department of Psychology, University of Oslo, Norway; c Department of Population and Public Health Sciences, University of Southern California, USA; d NORMENT, Institute of Clinical Medicine, University of Oslo, Norway; e Department of Psychiatric Research, Diakonhjemmet Hospital, Oslo, Norway; f Institute of Psychology, Leiden University, The Netherlands; g Leiden Institute for Brain and Cognition, Leiden University, The Netherlands; h Department of Psychiatry, University of Pittsburgh, USA

**Keywords:** Adolescence, Cortex, Gray matter, Longitudinal, Subcortical, White matter

## Abstract

A fundamental task in neuroscience is to characterize the brain’s developmental course. While replicable group-level models of structural brain development from childhood to adulthood have recently been identified, we have yet to quantify and understand individual differences in structural brain development. The present study examined inter-individual variability and sex differences in changes in brain structure, as assessed by anatomical MRI, across ages 8.0–26.0 years in 269 participants (149 females) with three time points of data (807 scans), drawn from three longitudinal datasets collected in the Netherlands, Norway, and USA. We further investigated the relationship between overall brain size and developmental changes, as well as how females and males differed in change variability across development. There was considerable inter-individual variability in the magnitude of changes observed for all examined brain measures. The majority of individuals demonstrated decreases in total gray matter volume, cortex volume, mean cortical thickness, and white matter surface area in mid-adolescence, with more variability present during the transition into adolescence and the transition into early adulthood. While most individuals demonstrated increases in white matter volume in early adolescence, this shifted to a majority demonstrating stability starting in mid-to-late adolescence. We observed sex differences in these patterns, and also an association between the size of an individual’s brain structure and the overall rate of change for the structure. The present study provides new insight as to the amount of individual variance in *changes* in structural morphometrics from late childhood to early adulthood in order to obtain a more nuanced picture of brain development. The observed individual- and sex-differences in brain changes also highlight the importance of further studying individual variation in developmental patterns in healthy, at-risk, and clinical populations.

## 1. Introduction

Longitudinal MRI research conducted over the past two decades has demonstrated that the human brain undergoes a prolonged course of development, with changes in morphometry observed in the cortex, as well as in white matter and subcortical structures, throughout childhood and adolescence (Aubert-Broche et al., 2013; Goddings et al., 2013; Lebel and Beaulieu, 2011; Mutlu et al., 2013; Raznahan et al., 2011; Reynolds et al., 2019; Vijayakumar et al., 2016; Wierenga et al., 2014a,b; Wierenga, Bos, et al., 2018). While this research has had a pro-found impact on our understanding of brain development, most of these studies have focused on estimating group-level trajectories, and quantifying the degree of individual variability in structural brain development remains a neglected area of research (Becht and Mills, 2020). Characterizing variability across individuals in how the brain *changes* during development is needed to address some of the most pressing questions in developmental neuroscience. It is only with this knowledge that we can identify individuals who begin to deviate in neurotypical development, or tailor prevention and intervention efforts to impact the processes that are changing the most during different developmental periods.

A goal of developmental neuroscience is to define patterns of brain maturation. We know that, for instance, the cerebral cortex decreases in volume and thickness across the second decade of life before beginning to stabilize in the early twenties, and that cerebral white matter increases until some point in mid-to-late adolescence (Aubert-Broche et al., 2013; Mills et al., 2016; Tamnes et al., 2017; Wierenga, Langen, Oranje, et al., 2014). But the age at which these structures begin to stabilize—a measure of maturity for structural brain measures—likely varies across individuals. Subcortical structures also show heterogeneity in their volumetric development (Herting et al., 2018; Ostby et al., 2009; Wierenga, Langen, Ambrosino et al., 2014), and our previous work has demonstrated that the amygdala and nucleus accumbens vary in when they reach a point of maturity across individuals (Mills et al., 2014). Identifying the periods of development when there is more inter-individual variability in change vs. stability can inform theories on how immaturity vs. maturity in a given brain measure reflects cognitive, emotional, and behavioral processes across development (e.g. the imbalance model; Casey et al., 2008).

Several neurodevelopmental models of psychopathology hypothe-size deviations in the rate of brain development for individuals at risk for, or with already developed, mental health disorders (Shaw et al., 2010). Neuroimaging studies have indeed demonstrated that rates of change in structural brain development relate to psychopathological symptoms (Bos, Peters, et al., 2018; Bos, Wierenga, et al., 2018; Ducharme et al., 2014; Muetzel et al., 2018; Whittle et al., 2020). Notably, sex differences are also present in patterns of structural brain changes across childhood and adolescence (Herting et al., 2014, 2018; Wierenga, Bos, et al., 2018; Wierenga, Sexton, et al., 2018), which may be pertinent to understanding sex differences in onset, prevalence, and progression of psychopathology (Paus et al., 2008; Shaw et al., 2010). Currently, there are several large-scale initiatives that aim to identify the genetic and environmental factors that shape the developmental course of the brain (e.g. ABCD, IMAGEN, Generation R). Moving forward, however, these investigations of brain development would benefit from knowing the periods of development when the most inter-individual variability is likely to occur, and which measures of the brain demonstrate the most inter-individual variability in developmental change.

There are now several longitudinal MRI datasets of structural brain development that can be used to examine variability in brain change over time (Vijayakumar et al., 2018). Indeed, individual variability in how the brain develops can only be examined with longitudinal datasets. While large cross-sectional samples are able to quantify the normative range of values of a given brain measurement at different periods of development, only longitudinal datasets can quantify the normative range of how brain measurements change within individuals at different periods of development, as it is not possible to estimate average rates of change in a given developmental period with only one measurement collected per individual. Recently, we have conducted secondary analyses of multiple longitudinal datasets with two or more time points of data per individual to establish replicable group-level models of typical structural brain development from childhood to early adulthood (Herting et al., 2018; Mills et al., 2016; Tamnes et al., 2017). The focus of the present investigation is to characterize inter-individual variability and sex differences in *changes* of brain structure (morphometry) across development. We do so by taking advantage of three separate datasets of developing individuals with three time points of data, which is necessary to model individual-level slopes in a multi-level analysis framework.

While the main focus of the present study is characterizing inter-individual variability in structural brain changes, we also examine whether individuals who have higher or lower measurements of a given structure compared to similar-aged peers show different maturation patterns. This secondary focus of the present investigation is motivated by a common inference in the neuroimaging literature that the maturity of a developing individual’s brain size can be assessed by comparing them cross-sectionally to similar-aged peers. For example, children and adolescents with thinner cortex than similar-aged peers are often inferred as more mature, or faster-maturing, in their brain development (e.g., Paulus et al., 2019; Tamnes et al., 2018; Thijssen et al., 2020). In this example, the inference stems from the group-level observation that the cortex of the human brain decreases in thickness across childhood and adolescence, but fails to consider the large amount of variability neurotypically developing children can have in one given brain measure (Wierenga et al., 2019). For example, one child can have an average cortical thickness that is 85% of the value of another child the same age (from data presented in Tamnes et al., 2017). If the claim that children and adolescents with thinner cortex are more mature, we would expect that they would show less overall change in cortical thickness, as a lack of change in brain structure is one way to assess the maturity of the brain. We test this hypothesis, as well as the relationship between overall values of other structural brain measurements in an individual with observed changes during development. We also examine how the relationship between overall brain size and maturation patterns differ between females and males.

## 2. Methods

### 2.1. Participants

**Fig. 1.**
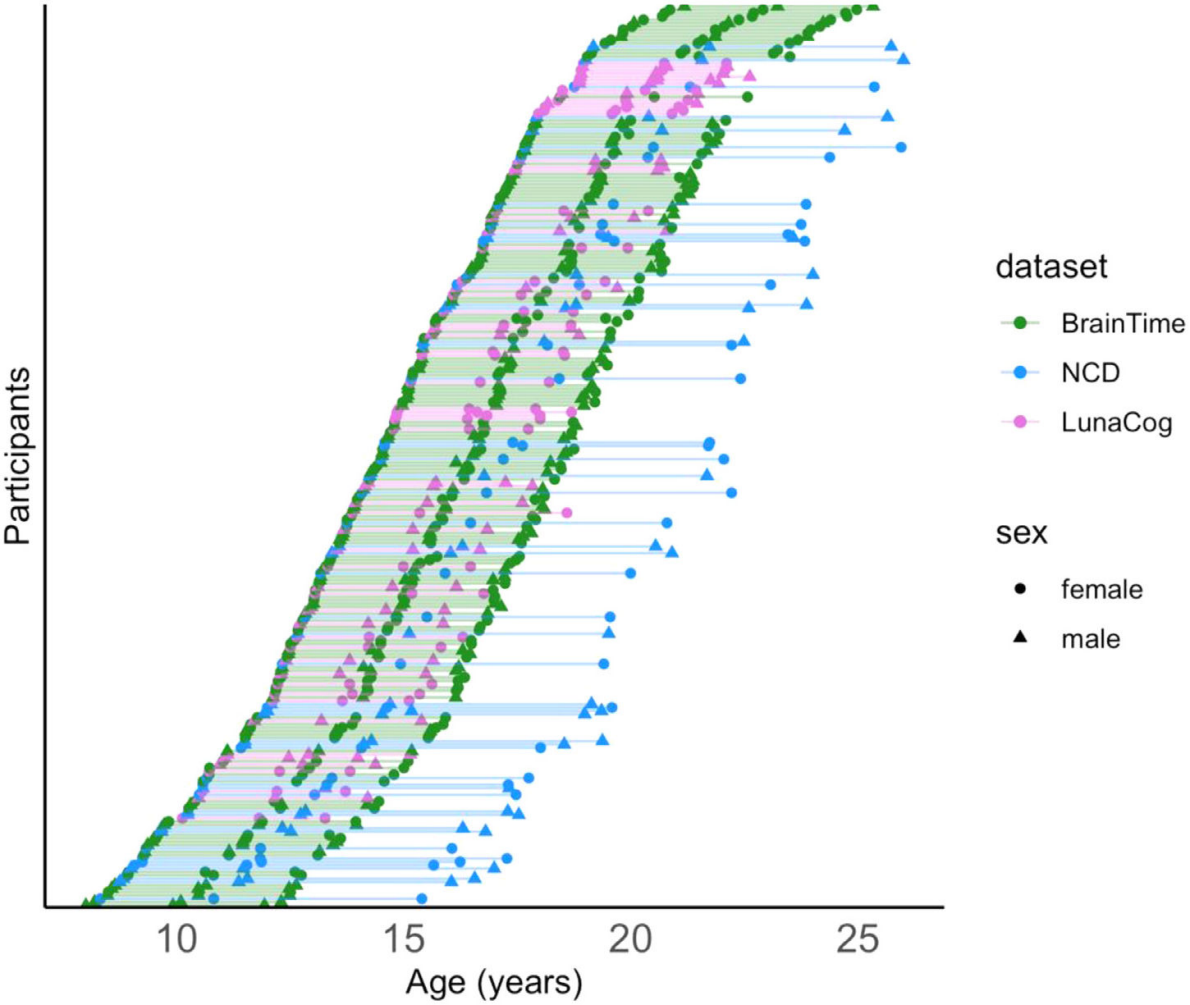
Scatter plot of age at scan for all participants. Each of the participants are shown in a different row, with each line connecting their three respective scans. Female (circle) and males (triangles) are denoted for each dataset (BrainTime: green; NCD: blue; LunaCog: Purple).

This study examined participants from three separate longitudinal datasets collected from independent research sites located in three countries: Leiden University (BrainTime), University of Oslo (Neurocognitive Development; NCD), and University of Pittsburgh (LunaCog). Only participants with three high-quality anatomical brain scans were included in the present analysis, for a total of 269 participants (149 females, 120 males). Demographic characteristics for each sample are described in Table 1, and the sampling design is illustrated in Figure 1. The distribution of ages varied slightly by dataset, with the average age of participants at first and last visit (and the interval between them) as follows: approximately 15–19 years (4 years) for BrainTime, 14–21 years (7 years) for NCD, and 15–18 years (3 years) for LunaCog. Scan intervals differed between datasets *X*^*2*^(2) = 579.74, *p* < 0.0001, with a mean interval of 2.23 ± 0.8 years. The number of data points included from females and males for each age (rounded to year) is illustrated in SFig. 1. Details regarding participant recruitment at each site are described in the Supplemental Material.

### 2.2. Image processing

Participants in the BrainTime and LunaCog samples were scanned using 3-T MRI machines, while the NCD sample was scanned using a 1.5-T MRI machine. Details regarding image acquisition at each site are described in the Supplemental Material. MRI processing was performed with the FreeSurfer 6.0 image analysis suite, which is documented and freely available online (http://surfer.nmr.mgh.harvard.edu/), on workstations and operating systems at their respective universities (see Supplemental Material). The technical details of these procedures are described in detail in seminal publications (Dale et al., 1999; Fischl et al., 1999, 2002). This processing stream includes motion correction (Reuter et al., 2010), removal of non-brain tissue using a hybrid watershed/surface deformation procedure (Ségonne et al., 2004), automated Talairach transformation, non-parametric non-uniform intensity normalization (Sled et al., 1998), tessellation of the gray/white matter boundary, automated topology correction (Fischl et al., 2001; Ségonne et al., 2007), and surface deformation following intensity gradients to optimally place the gray/white and gray/cerebrospinal fluid borders at the location where the greatest shift in intensity defines the transition to the other tissue class (Dale et al., 1999; Dale and Sereno, 1993; Fischl and Dale, 2000). Each cortical model was registered to a spherical atlas using individual cortical folding patterns to match cortical geometry across participants (Dale et al., 1999).

Images were then processed using FreeSurfer 6.0′s longitudinal stream (Reuter et al., 2012). This process includes the creation of an unbiased within-participant template space and image using robust, inverse consistent registration (Reuter et al., 2010). Several processing steps, such as skull stripping, Talairach transforms, atlas registration as well as spherical surface maps and parcellations are then initialized with common information from the within-participant template, significantly increasing reliability and statistical power (Reuter et al., 2012). All images were assessed for quality, as described further in the Supplemental Material.

### 2.3. Brain measures of interest

Measures of brain structure were computed at each time-point for each participant. For the purposes of this study, we included global measures of total gray matter volume, cortex volume, mean cortical thickness, white matter surface area, cerebral white matter volume, subcortical gray matter volume, as well as volumes of specific subcortical structures: amygdala, hippocampus, thalamus, pallidum, caudate, and putamen. We chose not to report on the nucleus accumbens of the FreeSurfer output, given less information about the test-retest reliability of the nucleus accumbens using the FreeSurfer longitudinal pipeline (Reuter et al., 2012).

### 2.4. Analysis procedure

The first aim of the study was to characterize inter-individual variability in structural brain change from late childhood into early adulthood. Each participant in the current analysis had three good-quality MRI scans, which allowed us to examine individual-level change across these three time points. We took two different approaches to measure inter-individual variability in change, which are described below.

First, we calculated change in a structure between each observation period, which gave us two observations of change per participant given that each participant had three time points of data. We then calculated the annualized change score by dividing the amount of change observed between time points by the amount of time between the two observation periods. We also calculated the annualized *percent* change, to provide an assessment of how a given structure changed relative to the overall size of the structure. We calculated the amount of change relative to the average size of the brain structure between observation periods. Specifically, this is how we calculated the annualized percent change for each structure for each of the two observation periods, with (*x* = observation):

$$\frac{\left(\left(\frac{\left(Brainmeaurement_{x+1}-Brainmeasurement_{x}\right)}{\left(Brainmeasurement_{x}+Brainmeasurement_{x+1}\right)}\right)\times 100\right)}{Age_{x+1}-Age_{x}}$$

Our rationale for examining both annualized change and annualized percent change is so that we could assess if there were any notable differences between the two, as we have done in our previous work (Mills et al., 2016).

For this first aim, we applied a generalized additive mixture model (GAMM; R package *mgcv* version 1.8–31 (Wood, 2011)) to visualize the group-level developmental pattern of change across the age period, as well as to assess if these patterns differed between females and males. GAMM allows us to flexibly model the group-level developmental pattern of change across the age period while nesting within participant, without assuming a given shape of the relationship between age and change. As opposed to polynomial-based linear mixed models, GAMM replaces the linear slope parameters with ‘smooth’ functions to find the optimal functional form between the predictor and response (Jones & Almond, 1992). The inclusion of site as a random factor had a negligible impact on models. LR tests comparing models with and without site as a random factor demonstrated that they were close to equivalent (p-values > .90). Thus, site was not included as a random factor in our final models. To assess sex differences, we compared three GAM models: an age only model, a model including a main effect of sex and age, and a model including an interaction between sex and age. We compared these three models using Akaike Information Criterion (AIC) and likelihood ratio statistics (LR test) to avoid overfitting, selecting the model with the lowest AIC score that was significantly different from the more parsimonious models. To formally compare the relationship between the magnitude of inter-individual variability of a given brain measure across age, we applied a generalized additive model with penalized cubic regression splines predicting the standard deviation of annualized percent change calculated within yearly age bins.

To examine inter-individual variability in the direction of developmental change, we categorized observations as increasing in a given structure if their annualized percent change was equal to or greater than the standard deviation of the annualized percent change observed in that structure calculated across the entire sample, decreasing if their annualized percent change was equal to or lesser than the negative value of the standard deviation, and stable if between these values. To formally compare the direction of inter-individual variability of a given brain measure across age, we performed chi-square tests comparing the number of observations of each direction of change across developmental period-defined age bins. For the purposes of these tests, if the age at the midpoint of the observation period was less than or equal to 13 years, the observation was classified as “transition into adolescence.” If the age at the midpoint of the observation period was between 14–18 years, the observation was classified as “mid-adolescence.” If the age at the midpoint of the observation period was greater than 18 years, the observation was classified as “transition into early adulthood.”

The second aim of the study was to examine the relationship between the size of an individual’s brain measure to their rate of change for that measure. For example, we wanted to assess if individuals with thicker cortex show a greater rate of change in cortical thickness over time as compared to individuals with thinner cortex. This is also accomplished in a GAMM, using a natural cubic spline to model the known non-linear age associations at the population-level, across the age range studied here. To measure the size of each examined structure for a given individual, we took the average of the value of that structure across all three observations. Studying the association between an individual’s average measure with their rate of change (slope) avoids a negative bias of the association estimate that measurement error can introduce when examining the initial measurement and subsequent measurements (for details see Chiolero et al., 2013). Thus, to answer the question if an individual’s rate of change in cortical thickness is associated with their cortical thickness (averaged across observations), then our modeling strategy had to take into account where the individual is relative to the population, or group-level, mean as seen with age and sex. For example, we know cortical thickness decreases over mid to late adolescence. Thus, an older adolescent (i.e. age 17 years) is likely to have thinner cortex than a younger adolescent (i.e. age 11 years). And for brain measures such as gray matter volumes, females are likely to have smaller gray matter volumes than males. Thus, our approach had to include understanding an individual’s average brain measure relative to the population averages seen for the individual’s age and sex. This was captured with the natural cubic spline.

With these considerations in mind, we applied a multi-level model of brain development as a function of age, with a participant-specific random intercept and slope. This is the same as fitting a hierarchical model for a given brain measurement (level 1) and slope (level 2). In level 1, the brain measure (i.e. cortical thickness) is modeled as a natural cubic spline with 4 degrees of freedom and an *intercept* that varies by sex and dataset. Importantly, a natural cubic spline is a piecewise cubic polynomial that ultimately allows for capturing nonlinearity in the data with constraints in place to reduce the likelihood of overfitting. In level 2, the *slopes* vary by sex and dataset and the deviation of the individual participant’s brain measure (i.e. an individual’s average cortical thickness) from the population mean for the measure (i.e. average cortical thickness). Specifically,

$${\text{Level1 : Y}}_{\text{ij}}=b_{1}+b_{2}X_{2}+b_{3}X_{3}+b_{4}X_{4}+b_{5}X_{2}X_{4}+b_{6}X_{3}X_{4}+b_{7i}{\text{age}}_{\text{ij}}\;\;+f({\text{age}}_{\text{ij}})+b_{0i}+e_{\text{ij}}$$

$${\text{Level2 : b}}_{7i}=g_{1}+g_{2}X_{2}+g_{3}X_{3}+g_{4}X_{4}+g_{5}X_{2}X_{4}+g_{6}X_{3}X_{4}\;\;+(g_{7}+g_{8}X_{4})({\text{avgY}}_{i}.-\text{avgY}..)+e_{\;i}^{s}$$

Where Y_ij_ is the brain measure and age_ij_ is age at the j^th^ visit (i.e. 1-3) for the i^th^ participant; b_1_ – b_6_ are coefficients that capture separate intercepts of females and males in each study (BrainTime, NCD, and LunaCog); b_7i_ is the individual’s trend over age, and f(age_ij_), the remaining terms that model age as a natural cubic spline (Hastie et al., 2009); b_0i_ reflects the individual’s random intercept (centered by group average based on sex and dataset) and e^s^_i_ their (group-level centered) random slope; g_1_ – g_6_ capture the average slope of age in females and males in each dataset (BrainTime, NCD, LunaCog); g_7_ and g_8_ are the primary covariates of interest; plus error (e_ij_). Specifically, g_7_ measures the association between the individual’s slope (i.e., rate of change in cortical thickness, b_0i_) and the individual’s average brain measure (avgY_i_., i.e., individual’s average cortical thickness) relative to the population average for that brain measure (avgY., i.e., cortical thickness) in females; g_8_ reflects the difference in association between an individual’s slope and the individual’s average brain measure (relative to the population average) in males (vs. females). Each predictor variable is centered by subtracting the average value. This two-level model can be fit by substituting the b_1i_ from level 2 in the level 1 model. The variables are coded using two indicator variables for dataset (*X*_*2*_ = 1 if NCD, 0 otherwise; *X*_*3*_ = 1 if LunaCog, 0 otherwise), and one for sex (*X*_*3*_ = 1 if male, 0 if female).

## 3. Results

### 3.1. Inter-individual variability in structural brain change

**Fig. 2.**
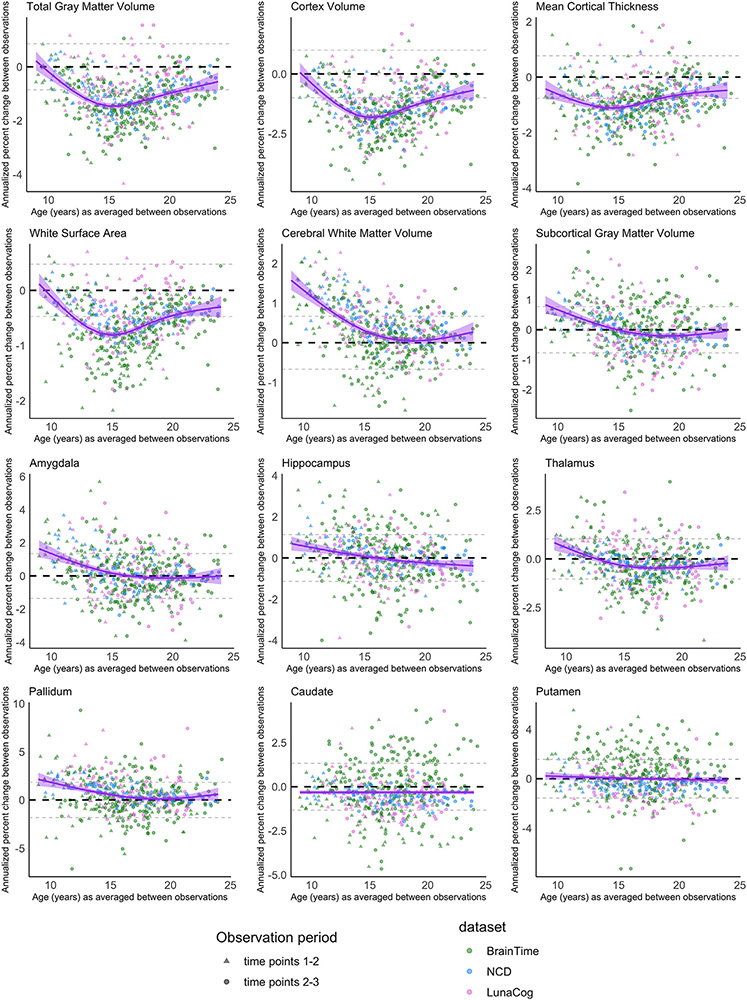
Annualized percent change over time for each individual (y-axis), against age at the midpoint of the observation period. The purple line reflects group-level annualized percent change seen with age. The black dashed line marks 0 on the y-axis and the dashed gray lines represent the standard deviation of annualized percent change across the whole sample. A) Total gray matter volume, cortex volume, cortical thickness, white surface area, and white matter volume B) Subcortical gray matter volume and specific subcortical structures. The equivalent graphs for annualized change can be seen in SFigure2.

**Fig. 3.**
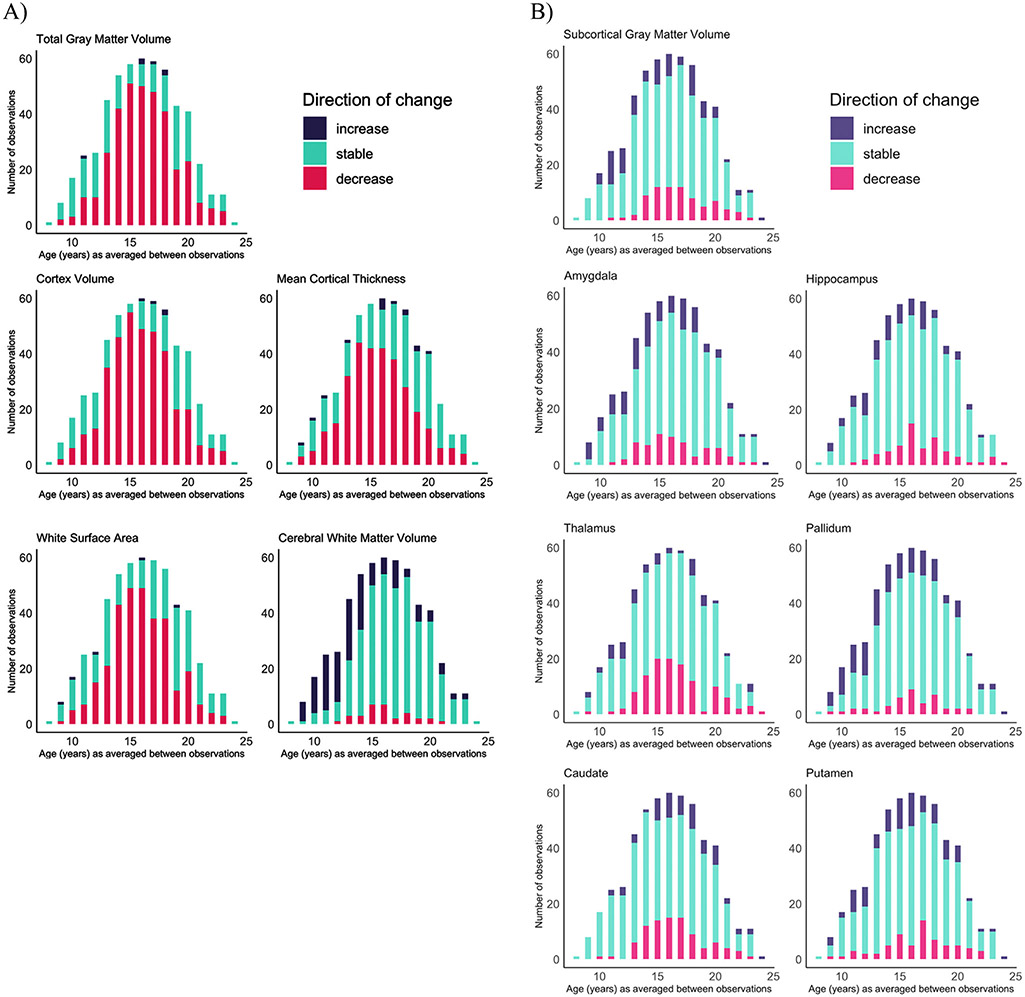
Number of individuals showing volume increases (purple), decreases (pink), or no change (turquoise) based on the individual’s age at the midpoint of observation period. A) Total gray matter volume, cortex volume, cortical thickness, white surface area, and white matter volume B) Subcortical gray matter volume and specific subcortical structures. See SFigure 3 for the same data graphed by percentage of individuals in each category.

**Fig. 4.**
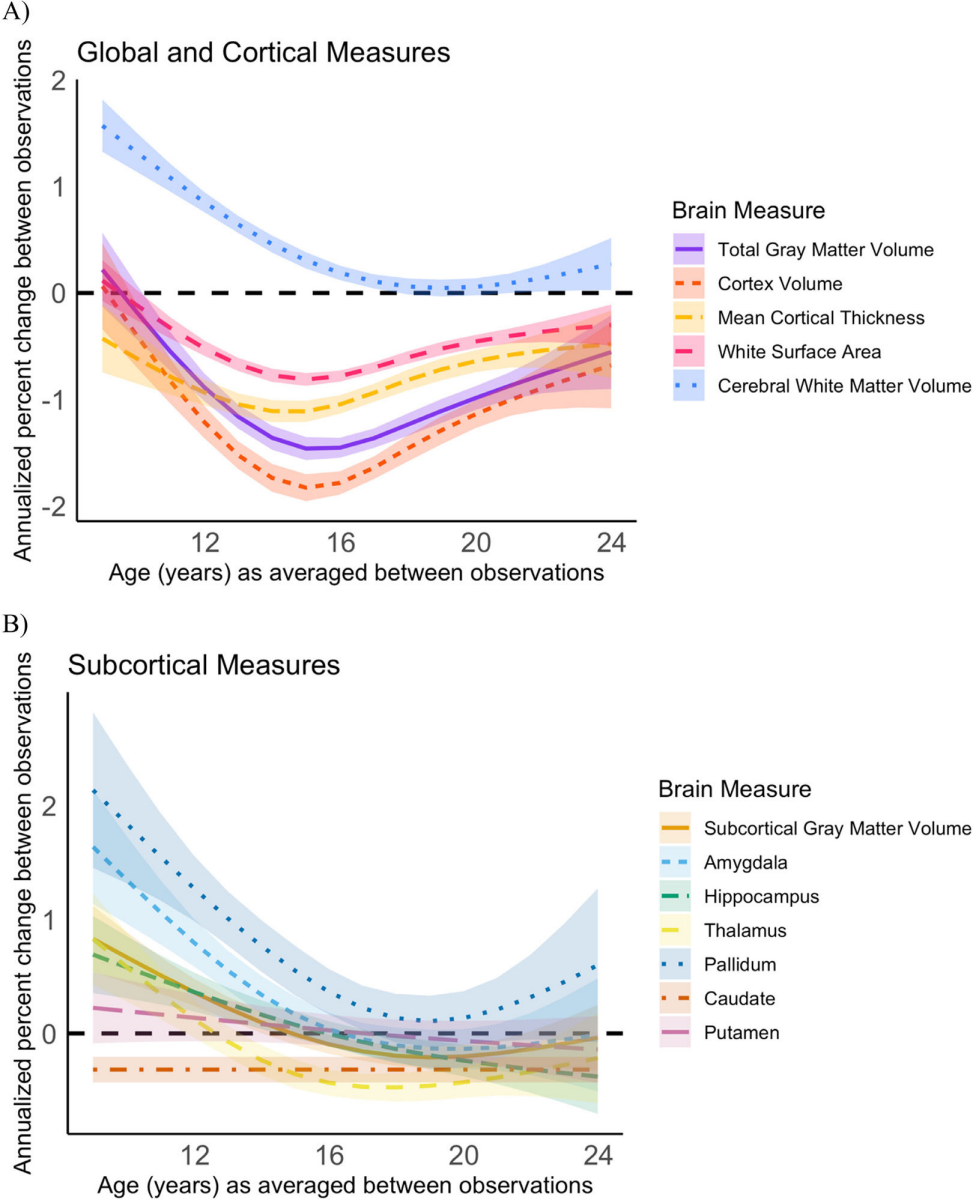
GAM models for annualized percent change for multiple brain measures together on one graph for the purpose of comparing group-level patterns of change. A) Total gray matter volume, cortex volume, cortical thickness, white surface area, and white matter volume B) Subcortical gray matter volume and specific subcortical structures.

We report the standard deviations for both the annualized change and annualized percent change across the sample for each structural brain measure in STable 1. Given that the annualized change and annualized percent change approaches resulted in almost identical developmental patterns of structural brain change (compare Fig. 2 with SFig. 2), we focus on describing the results from the annualized percent change measure since it allows for greater comparability across measures. For visualization, we plot annualized percent change against the age at the midpoint of observation period (Fig. 2). To visualize the number of observations categorized as either “decreasing” “stable” or “increasing” for each examined brain measure, we binned observations by yearly increments based on the individual’s age at the midpoint of observation period (Fig. 3). Our GAM models predicting annualized percent change by age are grouped together for visualization purposes in Fig. 4.

Inter-individual variability in direction and magnitude of change was present to some degree for every structural brain measure examined, and this variability was present across late childhood and into early adulthood (Figs. 2 and 3; SFig. 3). Similar developmental patterns of change were observed for total brain and cortical gray matter, mean cortical thickness, and white matter surface area. The majority of individuals demonstrated either stability or decreases in the transition into adolescence for cortical measurements and total gray matter volume, whereas in mid-adolescence (roughly between ages 14–17 years), the majority of individuals showed decreases in these measurements (see Table 2 for chi-square tests). By late adolescence and into early adulthood, there was again more variability, with the majority of individuals demonstrating either stability or decreases. These cortical measurements demonstrated the largest magnitude in decrease in change observed for most individuals in the early to mid-teens, and more stability observed in the early twenties (Figure 4). For cerebral white matter volume, the majority of individuals demonstrated increases in the transition into adolescence, shifting to the majority demonstrating stability by mid-to-late adolescence (*X*^*2*^ (2, 353) = 70.063, *p* < 0.0001). For subcortical gray matter volume and specific subcortical structures, variability in direction and magnitude of change was visible throughout the age-range investigated (STable 2; SFig. 3). For some structures, there appears to be subtle developmental patterns for directions of change, e.g. for the pallidum, most individuals demonstrated either an increase or stability in the transition into adolescence (*X^2^* (2, 353) = 29.661, *p* < 0.0001), and no substantial change from mid-adolescence onward (*X^2^* (2, 461) = 3.2101, *p* = 0.2009).

**Fig. 5.**
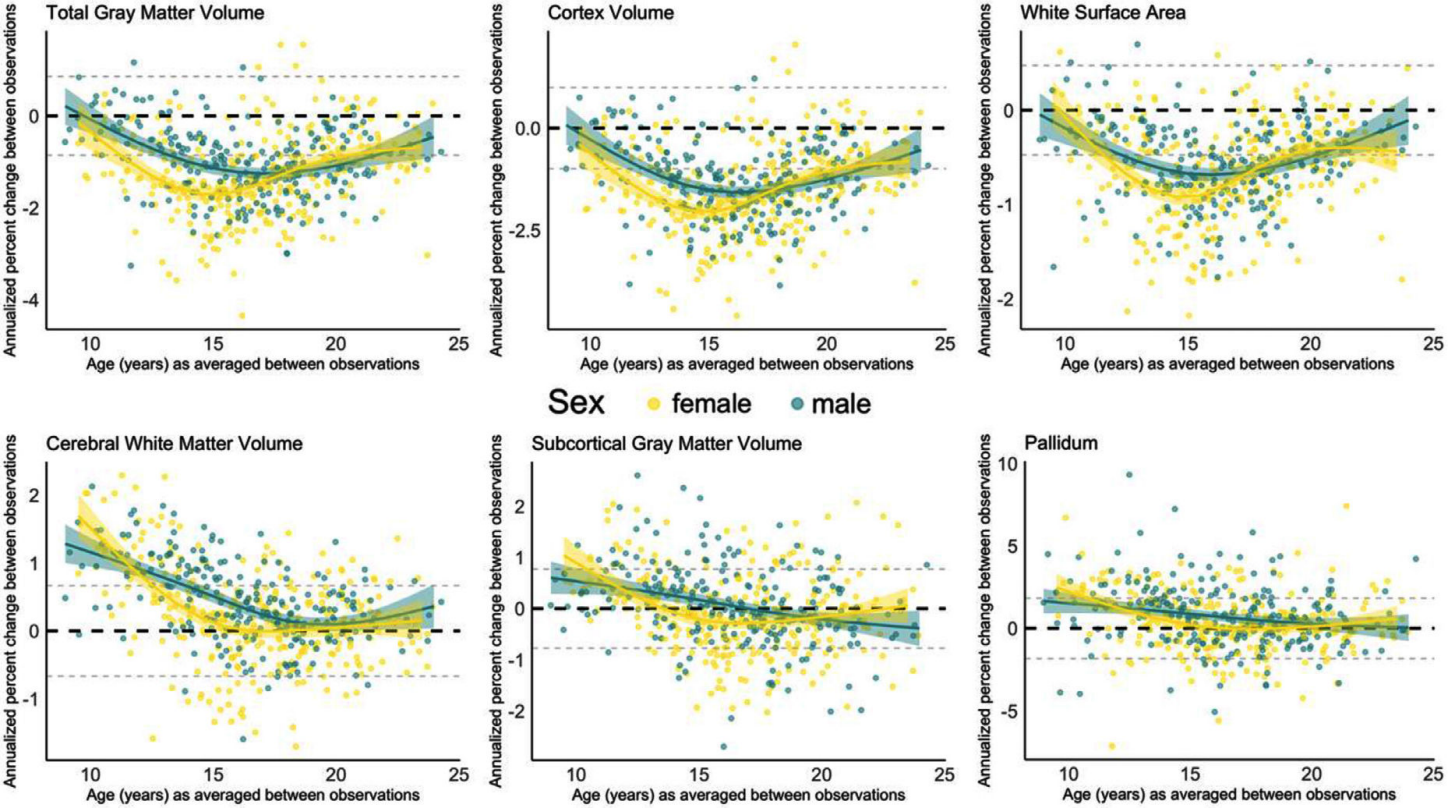
GAM models for annualized percent change for the brain measures with sex effects (see STable 3 for model comparison statistics). For total gray matter volume, white surface area, cerebral white matter volume, subcortical gray matter volume and pallidum, the best fit model included sex as a main effect. For cortex volume, the best fitting model included an interaction between sex and age. Female participants and their best fitting GAM model are represented in yellow, whereas males are represented in green.

We observed sex differences in the overall magnitude of annualized percent change for total gray matter volume, white surface area, cerebral white matter volume, subcortical gray matter volume, and for the pallidum, and we observed sex differences in the magnitude and pattern of annualized percent change for cortex volume (Fig. 5). The selected best fitting model did differ for two brain measures when change was assessed as annualized change instead of annualized percent change (see STable 3). For total gray matter volume, the model including sex and age as an interaction was the best fitting model when annual percent change was modeled by age, whereas this interaction model did not converge when annualized percent change was modeled by age. Further, the main effect of sex observed for white surface area when annualized percent change was modeled by age was not present when annual percent change was modeled by age. Model comparison statistics for both annualized change and annualized percent change measures are detailed in STable 3.

## 4. Relationship between an individual’s brain measure and rate of change

**Fig. 6.**
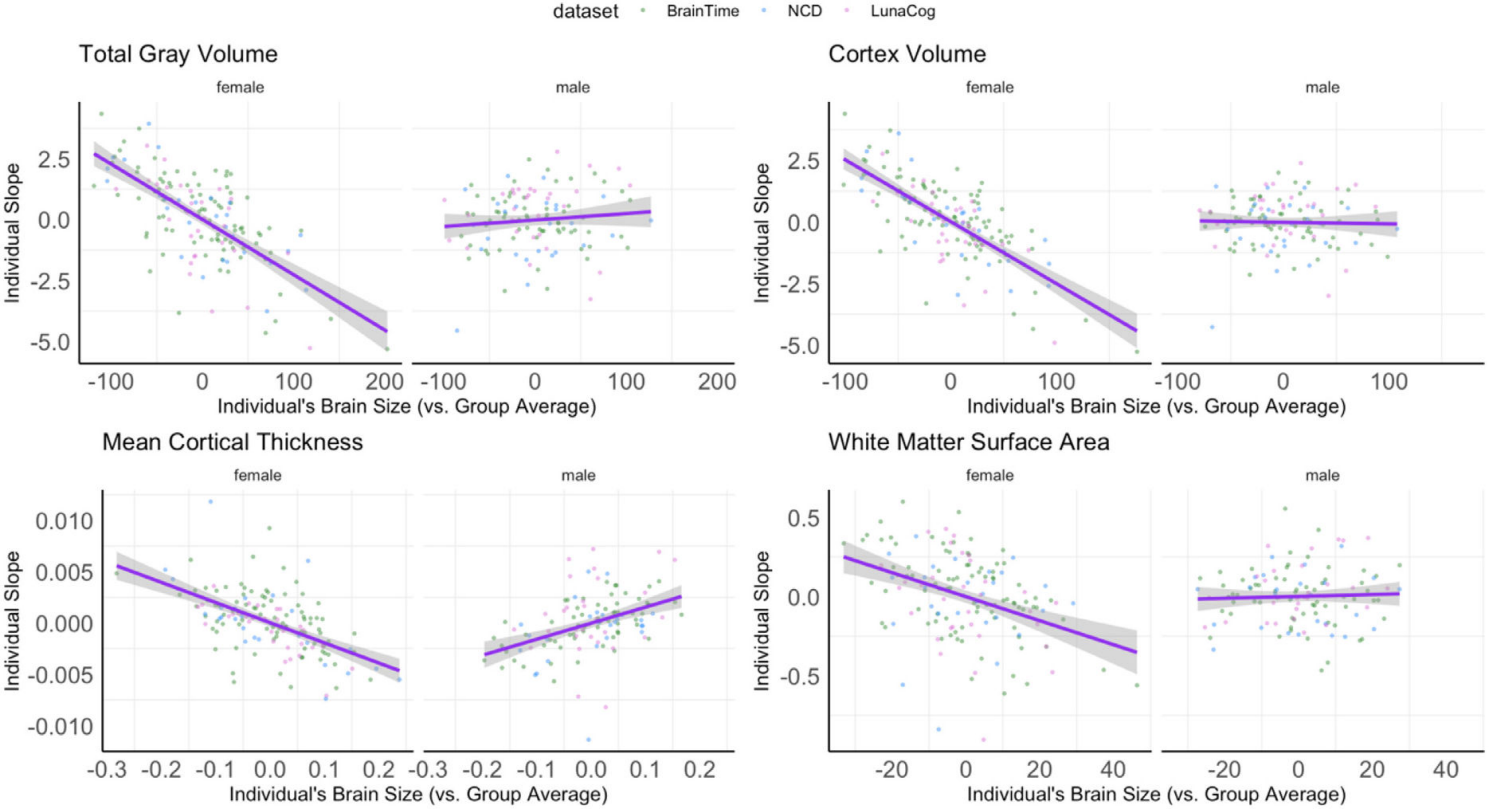
Significant sex differences in the association between individual’s slope as a function of an individual’s average brain measure compared to the group average (i.e. based on their study site and sex). Graphs present the magnitude of slope (i.e. rate of change) based on if an individual’s relative brain size to the group, with negative values reflecting smaller volumes and positive values reflecting larger volumes. Note, volume estimates have been scaled by 1000.

Population, or group-level, mean estimates for each brain measure are plotted by age and sex in SFig. 5. Results for the relationship between the rate of change of a given structural brain measure (i.e. change in white matter volume, change in amygdala volume, etc.) to an individual’s average brain measure (i.e. average white matter volume, average amygdala volume) and their sex are summarized in Table 3. Complete model outputs for each cortical and subcortical outcome can be found in STable 4. An individual’s total gray matter volume was found to significantly relate to an individual’s rate of change in gray matter volume over time and this was found to be different in females vs. males (Fig. 6). This same pattern was also seen for cortex volume, mean cortical thickness, and white matter surface area (Fig. 6). Females with larger brain measurements (i.e. gray matter, cortex volume, mean cortical thickness, white surface area) showed steeper decreases, or larger negative rates of change, in these brain outcomes (i.e. gray matter, cortex volume, mean cortical thickness, white surface area) as compared to females with smaller brain measurements. However, males showed similar rates of change in each outcome regardless of how large or small their volumes, cortical thickness, or white matter surface areas were. No relationship was seen between an individual’s rate of change and volumes of cerebral white matter, subcortical gray matter, or any of the specific subcortical structures examined in the present study (Table 3).

## 5. Discussion

The current collaborative research study utilized three longitudinal datasets including a total of 807 scans from 269 participants to quantify inter-individual variability in the development of whole brain, cortical, and subcortical measurements across 8.0 to 26.0 years of age. Inter-individual variability was present in each examined brain measurement, demonstrating that even when the majority of individuals follow a certain development pattern, some individuals will differ in the *direction* and *magnitude* of change. The majority of individuals demonstrated decreases in total gray matter volume, cortex volume, mean cortical thickness, and white matter surface area in mid-adolescence, with more variability present during the transition into adolescence and the transition into early adulthood. In contrast, the majority of individuals demonstrated increases in cerebral white matter in the transition into adolescence, with the majority showing stability starting in mid-adolescence and continuing into adulthood. Most individuals demonstrated little to no substantial change in subcortical structures or overall subcortical gray matter volume across the age range examined. Sex differences were largely reserved to cortical and whole brain measurements, in both our examination of patterns of annualized percent change across age, as well as in the association between brain structure size and rate of change.

Given that the majority of longitudinal development MRI studies in children and adolescents have been limited by two time points of data, most studies have looked at group-level effects of age. Few studies, however, have examined individual differences in how these structures change over time. The present study adds to the existing research by detailing just how variable changes in brain structure can be across late childhood and into young adulthood. Not only do individuals vary substantially in their overall brain structure size, but they can also differ in the magnitude, and sometimes direction, of observed developmental changes. We identified points in development where there is more inter-individual variability in direction of change for specific structural measurements, which can inform future work in several ways. For cortical measures, inter-individual variability in the direction of change was greatest in transition periods into adolescence and into early adulthood. Research that aims to examine linear developmental processes in cortical measurements may want to constrain their age range to the teen years as this is when we can have the highest confidence that most individuals will be showing the same direction of change. Furthermore, researchers might choose to examine cortical volume over cortical thickness or surface area for such an investigation, as this cortical measurement shows the most consistency in direction of change during the teen years. The current study can also inform research wishing to assess individual differences in “inflection points” or brain maturation. Notably, for cerebral white matter volume, the majority of variability was in regard to when an individual begins to show stability in that measurement.

Examining the relationship between an individual’s brain size and overall developmental change revealed differing patterns between females and males. For example, females with larger total gray matter and cortical volume, cortical thickness, and cortical surface area measurements also demonstrated larger decreases in those measurements across time. This association was not present in males. These findings provide additional evidence suggesting that not only do individual differences exist in the overall level and pattern of development (i.e. slope), but also that these two properties of individual differences in cortical brain development relate to one another in a region-specific fashion.

The observed individual-level and sex differences in brain *changes* from late childhood into young adulthood highlight the importance of further studying individual-level trajectories of brain maturation in cognitive and clinical neurodevelopmental investigations. A closer examination of each participant’s change over time suggests a complex interplay as to where the individual may fall relative to the group-average brain measurement. Regardlessof whether trajectories of structural brain development are linked to overall size for a given brain region, the amount of inter-individual variability seen among each of these structures demonstrates that prior work aimed at understanding risk for psychopathology between the sexes via group-level sex differences in rates of brain maturation may miss the mark in identifying which individuals go on to develop mental health problems.

### 5.1. Limitations

A substantial limitation to the current investigation is the lack of sociodemographic information available across the three longitudinal samples included in our analysis, which limits the ability to explore how potential environmental factors contribute to the variation seen in structural brain development and how this, in turn, may relate to behavior. Further, our definition of the threshold for what constituted a developmental change in a given brain measure represents a best estimate rather than a definitive rule. We calculated this threshold for each brain measure based on the overall standard deviation in annualized change (or annualized percent change) observed across the entire sample, which yielded estimates similar to those calculated from test-retest reliability studies of FreeSurfer measurements (Morey et al., 2010; Reuter et al., 2012). Nevertheless, our threshold for identifying change could have an impact on the conclusions of the present study and we suggest that future studies include multiple scans per individual in a given time point in order to best differentiate between time point measurement error from true developmental change. The current work also does not compare the inter-individual variability in developmental change across cortical regions—a key next direction to identify differences in when cortical regions undergo the most variable development between individuals. For example, frontal areas may be particularly relevant targets for future work, given evidence that rates of change in frontal regions vary in children and adolescents experiencing different levels of mental health symptoms (Ducharme et al., 2014; Bos et al., 2018), or different environmental experiences (Whittle et al., 2014). While the current work is not able to relate inter-individual variability in brain development to other developmental processes, future studies would benefit from relating these measures of changes to cognition, behavior, and mental health during adolescence. Finally, future work examining individuals with at least four time points of data would allow for estimation of non-linear individual slopes, which is more likely to resemble the shape of development for many structural brain measurements spanning the period of late childhood to early adulthood.

## 6. Conclusions

The present study demonstrates that individuals vary in the *direction* and *magnitude* of structural brain changes across late childhood into young adulthood. For cortical measurements, the greatest inter-individual variability in the direction of change was observed during transition periods into adolescence and into early adulthood. In contrast, the majority of individuals demonstrated increases in cerebral white matter in the transition into adolescence, with individuals starting to stabilize in mid-adolescence. The magnitude of changes observed differed between females and males for whole brain measurements. Inter-individual variability in rates of change related to overall brain size differently between females and males. Female participants demonstrated a negative relationship between brain size and change for cortical measurements, whereas this pattern was not seen in males.

## Data code and availability statement

The data used in the present study is derived from three studies from three separate laboratories. To access the data used in the present study, please contact the corresponding authors so that they can direct you to the appropriate individuals. The software used to process and analyze the data (FreeSurfer and R) are open source and freely available. The analysis script for the present study is freely available for all to use here: https://github.com/devbrainlab/variabilitybraindev

## Supplementary Material

1

2

3

## Figures and Tables

**Table 1:** Distribution of age and sex for the number of scans for each dataset. All participants had to have three good-quality MRI scans to be included in the present study.

	BrainTime		Neurocognitive Development (NCD)		LunaCog		Overall	
	Female (*N*=267)	Male (*N*=192)	Female (*N*=84)	Male (*N*=78)	Female (*N*=96)	Male (*N*=90)	Female (*N*=447)	Male (*N*=360)
**age**								
Mean (SD)	16.7 (3.58)	16.2 (3.44)	16.8 (4.26)	16.5 (4.41)	16.6 (2.82)	16.7 (3.13)	16.7 (3.56)	16.4 (3.60)
Median [Min, Max]	16.7 [8.50, 25.0]	16.5 [8.01, 25.3]	16.9 [8.33, 25.9]	16.5 [8.75, 26.0]	16.7 [10.1, 22.1]	16.9 [10.5, 22.6]	16.7 [8.33, 25.9]	16.6 [8.01, 26.0]

**Table 2:** Chi-square tests of age-bins and categorized direction of change. For the purposes of these tests, if the age at the midpoint of the observation period was less than or equal to 13 years, the observation was classified as “transition into adolescence.” If the age at the midpoint of the observation period was between 14-18 years, the observation was classified as “mid-adolescence.” If the age at the midpoint of the observation period was greater than 18 years, the observation was classified as “transition into early adulthood.”

*Measure*	Across age bins			≤13 vs. 14-18 years			14-18 vs. >18 years		
	*X^2^*	*df*	*p-value*	*X^2^*	*df*	*p-value*	*X^2^*	*df*	*p-value*
Total Gray Matter Volume	65.569	4	0.0000	60.657	2	0.00000	28.183	2	0.00000
Cortex Volume	78.748	4	0.0000	62.238	2	0.00000	52.978	2	0.00000
Mean Cortical Thickness	48.796	4	0.0000	18.553	2	0.00009	43.981	2	0.00000
White Surface Area	57.906	4	0.0000	37.409	2	0.00000	35.637	2	0.00000
Cerebral White Matter Volume	119.219	4	0.0000	70.063	2	0.00000	14.504	2	0.00071
Subcortical Gray Matter Volume	27.369	4	0.0000	26.543	2	0.00000	0.971	2	0.61538
Amygdala	27.055	4	0.0000	14.917	2	0.00058	7.04	2	0.02960
Hippocampus	18.736	4	0.0009	7.993	2	0.01838	6.82	2	0.03304
Thalamus	32.24	4	0.0000	29.14	2	0.00000	6.599	2	0.03690
Pallidum	45.775	4	0.0000	29.661	2	0.00000	3.21	2	0.20088
Caudate	23.964	4	0.0001	18.556	2	0.00009	5.957	2	0.05087
Putamen	7.345	4	0.1187	5.057	2	0.07979	0.455	2	0.79651

**Table 3:** Generalized Additive Mixed-effects Models (GAMM) examining associations between participant’s slope and average brain size. Degrees of freedom, F-statistic, and p-value for Type III sum of squares are presented for each fixed effect of interest, as well as total observations and overall model fit (*R*^*2*^).

		Individual Slope by Brain Size						
Measure	Finding	Effect (in Females)			Sex Difference (vs. Male)		
		df	F	p	df	F	p	*R^2^*
Total Gray Matter Volume	Sex Interaction	1	26.43	**0.0000004**	1	12.79	**0.0004**	0.988
Cortex Volume	Sex Interaction	1	28.580	**0.0000001**	1	9.755	**0.002**	0.987
Mean Cortical Thickness	Sex Interaction	1	4.107	**0.0432**	1	6.718	**0.001**	0.924
White Surface Area	Sex Interaction	1	9.882	**0.002**	1	3.840	**0.051**	0.997
Cerebral White Matter Volume	None	1	1.056	0.305	1	0.577	0.448	0.996
Subcortical Gray Matter Volume	None	1	2.533	0.112	1	2.626	0.106	0.982
Amygdala	None	1	0.229	0.632	1	1.285	0.258	0.975
Caudate	None	1	0.508	0.476	1	0.122	0.727	0.972
Hippocampus	None	1	1.352	0.246	1	1.356	0.245	0.980
Pallidum	None	1	2.082	0.150	1	1.595	0.207	0.949
Putamen	None	1	0.006	0.940	1	0.047	0.828	0.956
Thalamus	None	1	0.002	0.964	1	0.017	0.896	0.980

## References

[R1] Aubert-Broche B, Fonov VS, García-Lorenzo D, Mouiha A, Guizard N, Coupé P, Eskildsen SF, Collins DL, 2013. A new method for structural volume analysis of longitudinal brain MRI data and its application in studying the growth trajectories of anatomical brain structures in childhood. Neuroimage 82, 393–402. doi:10.1016/j.neuroimage.2013.05.065. 23719155

[R2] Becht AI, Mills KL, 2020. Modeling individual differences in brain development. Biol. Psychiatry 88 (1), 63–69. doi:10.1016/j.biopsych.2020.01.027. 32245576 PMC7305975

[R3] Bos MGN, Peters S, van de Kamp FC, Crone EA, Tamnes CK, 2018. Emerging depression in adolescence coincides with accelerated frontal cortical thinning. J. Child Psychol. Psychiatry 59 (9), 994–1002. doi:10.1111/jcpp.12895. 29577280 PMC6120477

[R4] Bos MGN, Wierenga LM, Blankenstein NE, Schreuders E, Tamnes CK, Crone EA, 2018. Longitudinal structural brain development and externalizing behavior in adolescence. J. Child Psychol. Psychiatry 59 (10), 1061–1072. doi:10.1111/jcpp.12972. 30255501 PMC6175471

[R5] Casey BJ, Getz S, Galvan A, 2008. The adolescent brain. Develop. Rev 28 (1), 62–77. doi:10.1016/j.dr.2007.08.003. PMC2500212 18688292

[R6] Chiolero A, Paradis G, Rich B, Hanley JA, 2013. Assessing the relationship between the baseline value of a continuous variable and subsequent change over time. Front. Public Health 1, 29. doi:10.3389/fpubh.2013.00029. 24350198 PMC3854983

[R7] Dale AM, Fischl B, Sereno MI, 1999. Cortical surface-based analysis: I. segmentation and surface reconstruction. Neuroimage 9 (2), 179–194. doi:10.1006/nimg.1998.0395. 9931268

[R8] Dale AM, Sereno MI, 1993. Improved localizadon of cortical activity by combining EEG and MEG with MRI cortical surface reconstruction: a linear approach. J. Cogn. Neurosci 5 (2), 162–176. doi:10.1162/jocn.1993.5.2.162. 23972151

[R9] Ducharme S, Albaugh MD, Hudziak JJ, Botteron KN, Nguyen T-V, Truong C, Evans AC, Karama S Brain Development Cooperative Group, 2014. Anxious/depressed symptoms are linked to right ventromedial prefrontal cortical thickness maturation in healthy children and young adults. Cereb. Cortex 24 (11), 2941–2950. doi:10.1093/cercor/bht151. 23749874 PMC4193463

[R10] Fischl B, Dale AM, 2000. Measuring the thickness of the human cerebral cortex from magnetic resonance images. Proc. Natl. Acad. Sci 97 (20), 11050–11055. doi:10.1073/pnas.200033797. 10984517 PMC27146

[R11] Fischl B, Liu A, Dale AM, 2001. Automated manifold surgery: Constructing geometrically accurate and topologically correct models of the human cerebral cortex. IEEE Trans. Med. Imaging 20 (1), 70–80. doi:10.1109/42.906426. 11293693

[R12] Fischl B, Salat DH, Busa E, Albert M, Dieterich M, Haselgrove C, van der Kouwe A, Killiany R, Kennedy D, Klaveness S, Montillo A, Makris N, Rosen B, Dale AM, 2002. Whole brain segmentation: automated labeling of neuroanatomical structures in the human brain. Neuron 33 (3), 341–355. 11832223 10.1016/s0896-6273(02)00569-x

[R13] Fischl B, Sereno MI, Dale AM, 1999. Cortical surface-based analysis. II: Inflation, flattening, and a surface-based coordinate system. Neuroimage 9 (2), 195–207. doi:10.1006/nimg.1998.0396. 9931269

[R14] Goddings A-L, Mills KL, Clasen LS, Giedd JN, Viner RM, Blakemore S-J, 2013. The influence of puberty on subcortical brain development. Neuroimage doi:10.1016/j.neuroimage.2013.09.073. PMC3991320 24121203

[R15] Hastie T, Tibshirani R, Friedman J, 2009. Education and nonmarket outcomes. Springer.

[R16] Herting MM, Gautam P, Spielberg JM, Kan E, Dahl RE, Sowell ER, 2014. The role of testosterone and estradiol in brain volume changes across adolescence: a longitudinal structural MRI study. Hum. Brain Mapp 35 (11), 5633–5645. doi:10.1002/hbm.22575. 24977395 PMC4452029

[R17] Herting MM, Johnson C, Mills KL, Vijayakumar N, Dennison M, Liu C, Goddings A-L, Dahl RE, Sowell ER, Whittle S, 2018. Development of subcortical volumes across adolescence in males and females: A multisample study of longitudinal changes. Neuroimage 172, 194–205. 29353072 10.1016/j.neuroimage.2018.01.020 PMC5910239

[R18] Jones K, Almond S, 1992. Moving out of the linear rut: the possibilities of generalized additive models. Transactions of the Institute of British Geographers 434–447.

[R19] Lebel C, Beaulieu C, 2011. Longitudinal development of human brain wiring continues from childhood into adulthood. J. Neurosci 31 (30), 10937–10947. doi:10.1523/JNEUROSCI.5302-10.2011. 21795544 PMC6623097

[R20] Mills KL, Goddings A-L, Clasen LS, Giedd JN, Blakemore S-J, 2014. The developmental mismatch in structural brain maturation during adolescence. Dev. Neurosci 36 (3–4), 147–160. doi:10.1159/000362328. 24993606

[R21] Mills KL, Goddings A-L, Herting MM, Meuwese R, Blakemore S-J, Crone EA, Dahl RE, Guroglu B, Raznahan A, Sowell ER, Tamnes CK, 2016. Structural brain development between childhood and adulthood: convergence across four longitudinal samples. Neuroimage 141, 273–281. doi:10.1016/j.neuroimage.2016.07.044. 27453157 PMC5035135

[R22] Morey RA, Selgrade ES, Wagner HR 2nd, Huettel SA, Wang L, McCarthy G, 2010. Scan-rescan reliability of subcortical brain volumes derived from automated segmentation. Hum. Brain Mapp 31 (11), 1751–1762. doi:10.1002/hbm.20973. 20162602 PMC3782252

[R23] Muetzel RL, Blanken LME, van der Ende J, El Marroun H, Shaw P, Sudre G, van der Lugt A, Jaddoe VWV, Verhulst FC, Tiemeier H, White T, 2018. Tracking brain development and dimensional psychiatric symptoms in children: a longitudinal population-based neuroimaging study. Am. J. Psychiatry 175 (1), 54–62. doi:10.1176/appi.ajp.2017.16070813. 28817944

[R24] Mutlu AK, Schneider M, Debbané M, Badoud D, Eliez S, Schaer M, 2013. Sex differences in thickness, and folding developments throughout the cortex. Neuroimage 82, 200–207. doi:10.1016/j.neuroimage.2013.05.076. 23721724

[R25] Ostby Y, Tamnes CK, Fjell AM, Westlye LT, Due-Tønnessen P, Walhovd KB, 2009. Heterogeneity in subcortical brain development: A structural magnetic resonance imaging study of brain maturation from 8 to 30 years. J. Neurosci 29 (38), 11772–11782. doi:10.1523/JNEUROSCI.1242-09.2009. 19776264 PMC6666647

[R26] Paulus MP, Squeglia LM, Bagot K, Jacobus J, Kuplicki R, Breslin FJ, Bodurka J, Morris AS, Thompson WK, Bartsch H, Tapert SF, 2019. Screen media activity and brain structure in youth: Evidence for diverse structural correlation networks from the ABCD study. Neuroimage 185, 140–153. doi:10.1016/j.neuroimage.2018.10.040. 30339913 PMC6487868

[R27] Paus T, Keshavan M, Giedd JN, 2008. Why do many psychiatric disorders emerge during adolescence? Nat. Rev. Neurosci 9 (12), 947–957. doi:10.1038/nrn2513. 19002191 PMC2762785

[R28] Raznahan A, Shaw P, Lalonde F, Stockman M, Wallace GL, Greenstein D, Clasen L, Gogtay N, Giedd JN, 2011. How does your cortex grow? J. Neurosci 31 (19), 7174–7177. doi:10.1523/JNEUROSCI.0054-11.2011. 21562281 PMC3157294

[R29] Reuter M, Rosas HD, Fischl B, 2010. Highly accurate inverse consistent registration: a robust approach. Neuroimage 53 (4), 1181–1196. doi:10.1016/j.neuroimage.2010.07.020. 20637289 PMC2946852

[R30] Reuter M, Schmansky NJ, Rosas HD, Fischl B, 2012. Within-subject template estimation for unbiased longitudinal image analysis. Neuroimage 61 (4), 1402–1418. doi:10.1016/j.neuroimage.2012.02.084. 22430496 PMC3389460

[R31] Reynolds JE, Grohs MN, Dewey D, Lebel C, 2019. Global and regional white matter development in early childhood. Neuroimage 196, 49–58. doi:10.1016/j.neuroimage.2019.04.004. 30959194

[R32] Ségonne F, Dale AM, Busa E, Glessner M, Salat D, Hahn HK, Fischl B, 2004. A hybrid approach to the skull stripping problem in MRI. Neuroimage 22 (3), 1060–1075. doi:10.1016/j.neuroimage.2004.03.032. 15219578

[R33] Ségonne F, Pacheco J, Fischl B, 2007. Geometrically accurate topology-correction of cortical surfaces using nonseparating loops. IEEE Trans. Med. Imaging 26 (4), 518–529. doi:10.1109/TMI.2006.887364. 17427739

[R34] Shaw P, Gogtay N, Rapoport J, 2010. Childhood psychiatric disorders as anomalies in neurodevelopmental trajectories. Hum. Brain Mapp 31 (6), 917–925. doi:10.1002/hbm.21028. 20496382 PMC6870870

[R35] Sled JG, Zijdenbos AP, Evans AC, 1998. A nonparametric method for automatic correction of intensity nonuniformity in MRI data. IEEE Trans. Med. Imaging 17 (1), 87–97. doi:10.1109/42.668698. 9617910

[R36] Tamnes CK, Herting MM, Goddings A-L, Meuwese R, Blakemore S-J, Dahl RE, Güroğlu B. Raznahan A. Sowell ER. Crone EA. 2017. Development of the cerebral cortex across adolescence: a multisample study of inter-related longitudinal changes in cortical volume, surface area, and thickness. J. Neurosci 37 (12), 3402–3412. 28242797 10.1523/JNEUROSCI.3302-16.2017 PMC5373125

[R37] Tamnes CK, Overbye K, Ferschmann L, Fjell AM, Walhovd KB, Blakemore S-J, Dumontheil I, 2018. Social perspective taking is associated with self-reported prosocial behavior and regional cortical thickness across adolescence. Dev. Psychol 54 (9), 1745–1757. doi:10.1037/dev0000541. 30058815 PMC6110335

[R38] Thijssen S, Collins PF, Luciana M, 2020. Pubertal development mediates the association between family environment and brain structure and function in childhood. Dev. Psychopathol 32 (2), 687–702. doi:10.1017/S0954579419000580. 31258099 PMC7525116

[R39] Vijayakumar N, Allen NB, Youssef G, Dennison M, Yücel M, Simmons JG, Whittle S, 2016. Brain development during adolescence: a mixed-longitudinal investigation of cortical thickness, surface area, and volume. Hum. Brain Mapp doi:10.1002/hbm.23154. PMC6867680 26946457

[R40] Vijayakumar N, Mills KL, Alexander-Bloch A, Tamnes CK, Whittle S, 2018. Structural brain development: a review of methodological approaches and best practices. Develop. Cogn. Neurosci 33, 129–148. doi:10.1016/j.dcn.2017.11.008. PMC5963981 29221915

[R41] Whittle S, Simmons JG, Dennison M, Vijayakumar N, Schwartz O, Yap MBH, …, Allen NB, 2014. Positive parenting predicts the development of adolescent brain structure: a longitudinal study. Develop. Cogn. Neurosci 8, 7–17. doi:10.1016/j.dcn.2013.10.006. PMC6990097 24269113

[R42] Whittle S, Vijayakumar N, Simmons JG, Allen NB, 2020. Internalizing and externalizing symptoms are associated with different trajectories of cortical development during late childhood. J. Am. Acad. Child Adolesc. Psychiatry 59 (1), 177–185. doi:10.1016/j.jaac.2019.04.006. 31047992

[R43] Wierenga LM, Bos MGN, Schreuders E, vd Kamp F, Peper JS, Tamnes CK, Crone EA, 2018. Unraveling age, puberty and testosterone effects on subcortical brain development across adolescence. Psychoneuroendocrinology 91, 105–114. doi:10.1016/j.psyneuen.2018.02.034. 29547741

[R44] Wierenga LM, Bos MGN, van Rossenberg F, Crone EA, 2019. Sex effects on development of brain structure and executive functions: greater variance than mean effects. J. Cogn. Neurosci 31 (5), 730–753. doi:10.1162/jocn_a_01375. 30726177

[R45] Wierenga LM, Langen M, Ambrosino S, van Dijk S, Oranje B, Durston S, 2014. Typical development of basal ganglia, hippocampus, amygdala and cerebellum from age 7 to 24. Neuroimage 96, 67–72. doi:10.1016/j.neuroimage.2014.03.072. 24705201

[R46] Wierenga LM, Langen M, Oranje B, Durston S, 2014. Unique developmental trajectories of cortical thickness and surface area. Neuroimage 87, 120–126. doi:10.1016/j.neuroimage.2013.11.010. 24246495

[R47] Wierenga LM, Sexton JA, Laake P, Giedd JN, Tamnes CK Pediatric Imaging, Neurocognition, and Genetics Study, 2018. A key characteristic of sex differences in the developing brain: greater variability in brain structure of boys than girls. Cereb. Cortex 28 (8), 2741–2751. doi:10.1093/cercor/bhx154. 28981610 PMC6041809

[R48] Wood SN. 2011. Fast stable restricted maximum likelihood and marginal likelihood estimation of semiparametric generalized linear models. J. Royal Statist. Soc.: Series B (Statist. Meth.) 73 (1), 3–36.

